# The Sources of Chemical Contaminants in Food and Their Health Implications

**DOI:** 10.3389/fphar.2017.00830

**Published:** 2017-11-17

**Authors:** Irfan A. Rather, Wee Yin Koh, Woon K. Paek, Jeongheui Lim

**Affiliations:** ^1^Department of Applied Microbiology and Biotechnology, School of Biotechnology, Yeungnam University, Gyeongsan, South Korea; ^2^Food Technology Division, School of Industrial Technology, Universiti Sains Malaysia, Minden, Malaysia; ^3^National Science Museum, Ministry of Science, ICT and Future Planning, Daejeon, South Korea

**Keywords:** food contamination, chemical contaminants, pesticides, food control, toxins

## Abstract

Food contamination is a matter of serious concern, as the high concentration of chemicals present in the edibles poses serious health risks. Protecting the public from the degrees of the harmfulness of contaminated foods has become a daunting task. This article highlights the causes, types, and health implications of chemical contamination in food. The food contamination could be due to naturally occurring contaminants in the environment or artificially introduced by the human. The phases of food processing, packaging, transportation, and storage are also significant contributors to food contamination. The implications of these chemical contaminants on human health are grave, ranging from mild gastroenteritis to fatal cases of hepatic, renal, and neurological syndromes. Although, the government regulates such chemicals in the eatables by prescribing minimum limits that are safe for human consumption yet measures still need to be taken to curb food contamination entirely. Therefore, a variety of food needs to be inspected and measured for the presence of chemical contaminants. The preventative measures pertaining about the food contaminants problems are pointed out and discussed.

## Introduction

The phrase chemical contamination is a clear indication of the presence of chemicals where they should not be or are present in an amount that is in a higher concentration than the amount that is attributed as safe. The chemical hazards are one of the main causes of food contamination that associated with foodborne disease outbreaks (Faille et al., in press). The origins of chemical contaminants are various from the field to the plate, namely soil, environment, disinfection by-products, personal care products, air, water, and packaging material. Chemical contaminants inhibit almost all the mass-produced everyday use products such as disinfectants, plastics, detergents, deodorants, pesticides, and so on. Even the food that is consumed and the water that is taken is not safe from the invasion of chemicals in unsafe concentrations. Food contamination, whether accidental or intentional, is an unfortunate act that brings in its wake numerous serious implications on the human health. Food contamination has been recorded in history for as early as 8,000 years ago; however, the growth in agribusiness and globalizations have aided the problem in spreading all over the planet (Robertson et al., 2014). The US Centre for Disease Control and Prevention confirmed more than 11,000 foodborne infections in the year 2013 (Salter, 2014), with several agents like viruses, bacteria, toxins, parasites, metals, and other chemicals causing food contamination (Callejón et al., 2015). The symptoms of the foodborne illness due to chemical contamination range from mild gastroenteritis to fatal cases of hepatic, renal, and neurological syndromes. It is in this context that food contamination often breaks into the headlines as a result of its harmful consequences. A total of 1527 outbreaks of foodborne diseases were witnessed in the United States between 2009 and 2010, resulted in 29,444 illness cases and 23 deaths (CDC, 2013). Furthermore, food contamination has become more serious in recent years due to the development of industry and the consequent environmental pollution (Song et al., 2017). Besides that, the ingestion of contaminated food with pesticides and heavy metals could cause gastrointestinal infections (Song et al., 2017). For instance, an estimated 400 to 500 children died of acute lead poisoning due to ingestion of food contaminated with lead-contained soil and dust in Nigeria (Tirima et al., in press). Keeping such incidents in mind and the overall harmful health implications in the fore, this review examines the reasons and types of chemical contaminants in food along with individual's exposure to such contaminated foods on a daily basis and further elaborates the health impacts of such food impurities.

## The reasons for food contamination

Food is a crucial contributor to human health well-being and a major source of worry, pleasures, and stress (Wilcock et al., 2004), with one of the reasons behind the stress and worry, are the diseases caused as a result of contaminated food. There are multiple reasons for the contamination of food (Ingelfinger, 2008). Food preparation undergoes through a long chain of processing, where each stage is a potential source of chemical contaminants invasion of the food. Transportation of food can also lay the foundation for contamination of food, specifically under poor sanitary conditions (Unnevehr, 2000). Likewise, some chemicals are mixed deliberately during the food preparation process to improve the shelf life of a food product. The contaminants may include impurity food when cooked in the kitchen; nevertheless, the transmission is mainly dependent on the effectiveness of the kitchen hygiene though (Gorman et al., 2002). Chemical contaminants enter the food chain naturally as well with pathogens that are present in the environment and show high bacterial numbers on some key raw foods such as poultry meat (Humphrey et al., 2007).

## Types of food contaminants

Food contaminants typically include environmental contaminants, food processing contaminants, unapproved adulterants and food additives, and migrants from packaging materials (Mastovska, 2013). Environmental contaminants are impurities that are either introduced by human or occurring naturally in water, air or soil. Food processing contaminants include those undesirable compounds, which are formed in the food during baking, roasting, canning, heating, fermentation, or hydrolysis (Schrenk, 2004). The direct food contact with packaging materials can lead to chemical contamination due to the migration of some harmful substances into foods. Further, use of unapproved or erroneous additives may result in food contamination.

## Naturally occurring contaminants in food

Several bacteria, viruses, and parasites inhabit the surfaces of the raw food naturally. Contamination of raw food can also occur due to the sewage, soil, external surfaces, live animals, the internal organs of meat animals. An additional source of contaminated food is the food that originated from diseased animals although the health advancement has nearly eliminated this source of food contamination (Marriott and Gravani, 2006). Food contamination from the chemical sources includes the accidental mixing of chemical supplies in food or the chemicals in the animal feed or antibiotic injections given to poultry animals (Martin and Beutin, 2011). Several parasites are also present in the food by symbiotic relations between the organism and the parasite. Numerous of these cause foodborne infections and outbreaks. A broad categorizing of these parasites is presented in Table 1A (Newell et al., 2010).

**Table 1A:**
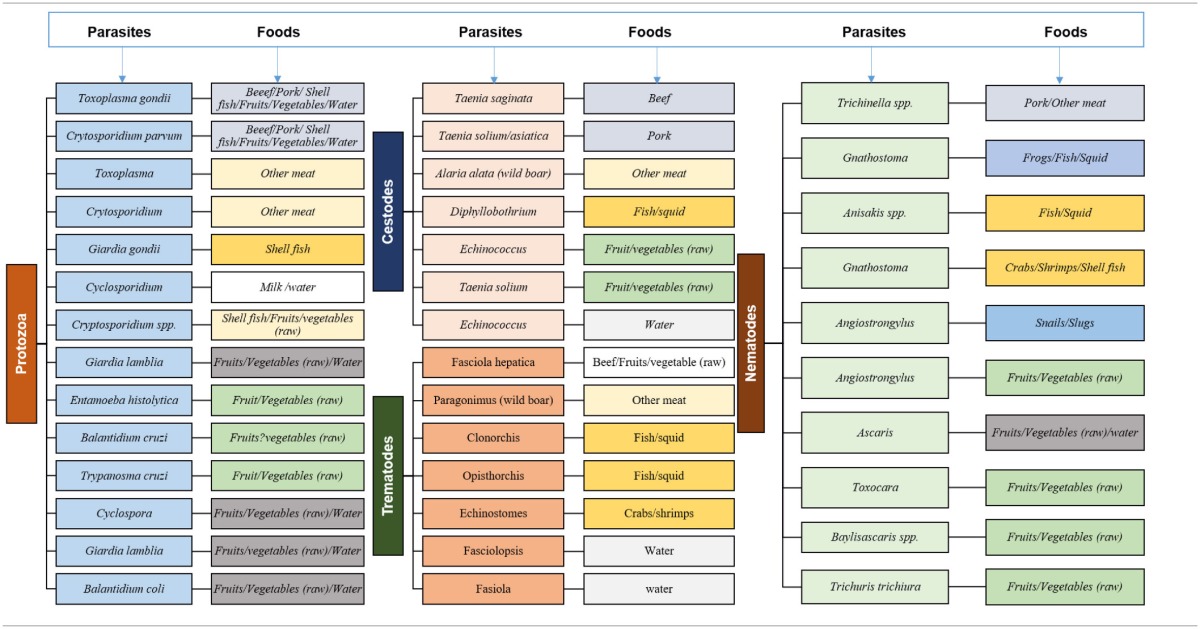
Parasites in different foods (modified and is being used with permission from Newell et al., 2010).

Enteric infections due to parasite can be transmitted via the fecal-oral route by consuming intrinsically the contaminated food or through the uptake of free-living parasites from the environments. Contamination of the food products such as meat, vegetables, and fruits is possible via the introduction of the parasite in the sewage, irrigation water, feces, soil, human handling or improper process of the infected meat. Food producing animals can itself transfer the parasites, as they are themselves infected (Pozio, 1998).

## Contamination during the food production, processing, storage, and preparation phases

Contaminants may be present in the food in their raw stages as a result of environmental sources of contaminants. During the transportation of food, common sources of contamination include the vehicle exhausts of diesel and petrol or cross-contamination in the vehicle being used for food transportation. Long-distance ships for transport are also often cross contaminated with chemicals used for disinfection or other sources (Nerín et al., 2007a). High barriers used for protection of food by wrapping it during long-distance transport are not always tested for their barrier properties, which makes it a cause of contamination. In the cleaning phase of food production and preparation, contaminants can invade due to the residues left from the disinfectants and cleaning agents on the surface of food handling equipment (Nageli and Kupper, 2006; Villanueva et al., 2017). Heating treatment in the production process is another source of contaminants. The use of high cooking temperature at homes and industries is the widely used method for food process. The use of high temperature for cooking paired with external factors potentially leads to the formation of toxic compounds that leave an impact on the food safety and quality. Toxic compounds such as nitrosamines chloropropanols, acrylamide, furanes, or PAHs are formed during the food processing methods like heating, roasting, grilling, baking, canning, fermentation, or hydrolysis (Nerín et al., 2016). Frying is a leading source of generation of a range of toxic compounds in the food preparation processes (Roccato et al., 2015). Additionally, microwave heating can also give birth contaminants in food, as the common feature of microwave cooking is that the food is cooked in the container or wrapping film (packaging material) in the microwave oven (Nerín et al., 2003). The microwavable packaging materials include paperboard, composites, and plastics and during the cooking components of these materials can transfer from the package to the food, resulting in a decline in food safety and quality (Ehlert et al., 2008).

**Figure 1:**
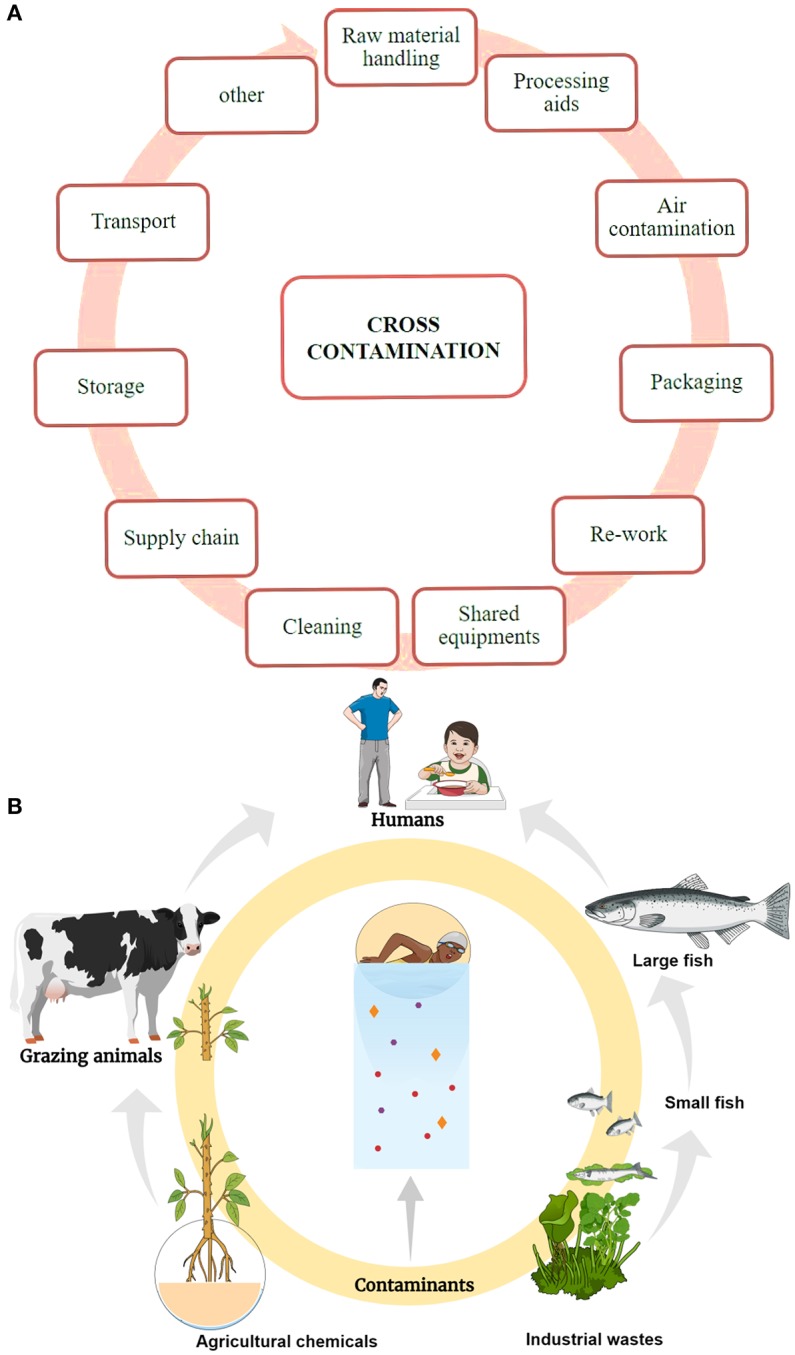
Food contamination. **(A)** Contamination in the food production and processing. **(B)** Contamination due to environmental influences.

Food packing carries several advantages like physical protection and enhanced food protection; however, it still can pose a threat (Marsh and Bugusu, 2007). Packaging processes make use of several additives like stabilizers, antioxidants, plasticizers, and slipping agents to improve the packaging material properties. Nevertheless, any direct or indirect contact with the food with the packaging material can result in the transference of these substances from the packaging into the food. Such a phenomenon is termed as migration. When metallic cans are used in packaging, corrosion stands as a source of food contamination due to the migration of metallic ions to food (Buculei et al., 2012). To avoid this, the inner side of cans are commonly coated with varnishes like epoxy resins to save from corrosion, but even the minor by-products from the epoxy resins manufacture like cyclo-di- BADGE, bisphenol A, or bisphenol A diglycidyl ether (BADGE) can migrate to food. Such compounds are known as endocrine disruptors (Cabado et al., 2008). There is also the risk of non-intentionally added substances migrating from the packaging material to the food producing adverse effects (Nerin et al., 2013). Food storage is another step that can lead to toxins in food. Some of the contaminating factors include direct sunlight that speeds deterioration of food and packaging and adsorption of unwanted off-odors. Foods with longer shelf life contain flavors and color that compromise with the nutritive value of food. Also, high fatty foods are prone to odor contamination (Nerín et al., 2007a). Food contamination due to the entire food processing to packaging stages is summed in Figure 1.

## Contamination due to environmental influences

The biosensor assay format helps to determine the numerous environmental pollutants that cause food contamination (Baeumner, 2003). Several metals, primarily toxic heavy metals cadmium, mercury, lead, and polychlorinated biphenyl (PCB) enter through the industrial environment to contaminate food. The instance of an industrial area of Huludao in Northeast China, which is seriously contaminated by heavy metals such as mercury, lead, cadmium, zinc, and copper due to the heavy metals smelting in the area (Zheng et al., 2007). Plants form the base of the food chain, and they can easily absorb toxic substances from the soil, contaminating not only fruits and vegetables but also the seafood (Peralta-Videa et al., 2009). The soil environment is another source of food contamination. Heavy metals from industrial areas can seep into the soil and enter into the food chain to infect the raw sources of food (Krishna and Govil, 2006). Pesticides used as plant protection agents also enter into the food chain and human exposure to these chemicals shows a wide array of health problems like immune suppression, diminished intelligence, hormone disruption, cancer, and reproductive abnormalities (Abhilash and Singh, 2009). Approximately 3 billion kg of pesticides is applied every year around the world, (Pimentel, 2005) which poses a serious threat, as the chemicals contaminate the raw sources of food. In the case of pesticides, however, the maximum residue level (MRL) is an important determinant of the risk it poses to human health. The pesticide residue levels in food are regulated by legislation to minimize its exposure to the consumer (Nasreddine and Parent-Massin, 2002). However, in numerous underdeveloped countries, such legislation is not in place or is poorly enacted. Similar to pesticides are the residues of veterinary drugs in the farm animals that may remain in the meat and threaten the individual through the exposure to these drug residues, transference of antibiotic resistance, and risk of allergies (Reig and Toldrá, 2008).

## Chemical contaminants in drinking water

The issue of food consumption has evolved from a short trading chain between producer and consumer to a complex chain of various parties (Pongratz et al., 2011). Similar to food, drinking water is also at a risk of contaminants with serious health implications not only for the human life but also for the marine life and other organisms that consume the impure water. The sources of these contaminants are multiple including industrial and municipal discharges, natural geological formations, urban and rural run-off, drinking water treatment process, and water distribution materials (Calderon, 2000). Human activities such as hydraulic fracturing and horizontal drilling have increased energy production, however, also increased the incidence of drinking water contamination. Drinking water sourced from groundwater can also be contaminated with heavy metals (e.g., nickel, mercury, copper, and chromium) which could resulted in increased cases of health defects of carcinogenic and noncarcinogenic nature (Wongsasuluk et al., 2013), including fecal contamination (Kostyla et al., 2015). Such a source of contamination of the drinking water is particularly prevalent in low and middle-income countries (Bain et al., 2014). By-products of pharmaceuticals are also toxic and another identified source of water contamination by chemicals (Shen and Andrews, 2011).

Drinking water contaminants include several chemicals such as arsenic, aluminum, lead, fluoride, disinfection by-products, radon, and pesticides (Table 1B). Their health effects range from numerous cancer, cardiovascular diseases, adverse reproductive outcomes, and neurological diseases. Currie et al. (2013) have also identified that the consumption of chemically contaminated water by mothers, specifically those who are less educated, show significant effects on the gestation of infants and birth weight of the baby.

**Table 1B:** Common chemical contaminants in drinking water reported in the recent literature.

**Chemical contaminants**	**Diseases/health effects caused**	**References**
Aluminum, arsenic	Skin, bladder, and prostate cancers, Alzheimer's and peripheral neuropathy, reproductive, cardiovascular, immunological, and neurological diseases	Barnaby et al., 2017; de Meyer et al., 2017
Disinfection by-products (trihalomethanes and dichloroacetic acid)	Leukemia, reproductive diseases, bladder, and colon cancers	Jeong et al., 2017; Villanueva et al., 2017
Fluoride	Osteosarcoma, skeletal fluorosis	Guissouma et al., 2017; Walia et al., 2017
Lead	Occupational cancers, haemoprotein degradation, intellectual disability, anti-social behavior, high blood pressure, heart disease, kidney disease, and reduced fertility	Rosen et al., 2017; Tirima et al., in press
Nitrate	Stomach, esophagus, bladder, brain, colon, rectum, pancreas, ovarian, and kidney cancers, adverse pregnancy outcomes, diabetes and thyroid disorders	Espejo-Herrera et al., 2015; Schullehner et al., in press
Pesticide residues (2, 4-D, malathion, diazinon, and fenpropimorph)	Leukemia, reproductive, immunological, and neurological cancers	Mekonen et al., 2016; Shi et al., 2018
Radon	Lung cancer	Gunnarsdottir et al., 2016; Jobbágy et al., 2017
Sulfate (gypsum, anhydrite, barite, and celestine)	Diarrhea, laxative effect	Călinescu et al., 2016; Song et al., 2017

## Health implications of food contaminants

Foodborne diseases number about 48 million illnesses annually in the US. (Gould et al., 2013) Chemically contaminated food has serious implications on the health of individuals. The harmful effects range from minor gastric problems to major health fatalities. Chemical contaminants are strongly linked with severe consequences, lack of personal control, and long-term effects (Kher et al., 2011). Food consumption is the most likely source of human exposure to metals. Metals such as cadmium and lead can easily enter the food chain. Heavy metals can seriously deplete specific nutrients in the body that can decline the immunological defenses, impair psycho-social facilities, and cause intrauterine growth retardation. Heavy metal consumption is also associated with malnutrition and increases the rates of gastrointestinal diseases (Khan et al., 2008). Food contaminants are also a leading cause of cancer (Abnet, 2007) Polychlorinated biphenyls (PCBs) exposure due to food contamination can adversely affect children's neurological development and the immune response (Schantz et al., 2004). Pesticides in the food as contaminants also show severe health implications. Excessive levels of these chemicals in the food cause neural and kidney damage, congenital disabilities, reproductive problems, and can prove to be carcinogenic (Bassil et al., 2007). The accumulation of pesticides in the tissues of the body can also result in metabolic degradation (Androutsopoulos et al., 2013). There is also the risk of neurodevelopmental disorders like attention deficit disorders, autism, cerebral palsy and mental retardation caused by industrial chemicals like arsenic, PCBs, and lead in both food and water. Exposure to such chemicals in the fetal stages of development can cause brain injury and such lifelong disabilities at much lower doses than those which can affect adult brain function (Grandjean and Landrigan, 2006).

## Individual exposure to food contaminants

Food consumption is a crucial pathway for exposure to contaminants from various sources. An individual's exposure to these contaminants is high, which accounts for the high number of hospitalized cases and illnesses not only in the US but also around the world. Food contaminants are in almost every foodstuff including fruit, baked goods, vegetables, poultry, meat, and dairy products (Kantiani et al., 2010). It is not uncommon for a single food item to contain residues of five or greater than five persistent chemical toxins (Schafer, 2002). A study examined the dietary exposure of 37 contaminants in the US and revealed that 20 of the studied contaminants had available cancer benchmark concentrations. These benchmark concentrations indicated that the daily exposure of the contaminants had a probability of showing adverse side effects (Dougherty et al., 2000). Another study estimated the exposure of numerous dietary contaminants on children; the results found that the cancer benchmark of the contaminants exceeded in all the children for dieldrin, arsenic, DDE, and dioxins (Vogt et al., 2012).

## The preventative measures to control food contamination

There is legislation in place to regulate the levels of several chemicals in the food. Unhealthy additives and adulterants are legally not allowed for use. However, effective surveillance and response systems are required to prevent chemical hazards from entering the food supply and posing harm to the public. The FDA prescribes the minimum levels of chemicals that are allowed in food, such as pesticide concentration should not go higher than the limit assigned (Bajwa and Sandhu, 2011). However, errors may still occur in following the determined concentration and guidelines. Particularly in the case of developing and underdeveloped countries, the legislation enforcement is still weak about administrating the concentration of harmful contaminants in the food. Some countries are highly dependent on agriculture, resulting in high levels of pesticides seeping into the ground water, contaminating both food and water. Non-regulated chemicals are of specific concern (Villanueva et al., 2013) and more research needs to focus on contaminants that escape human detection. Also, individual consumer concerns are essential as they can play a fundamental role in managing their health (Liang and Scammon, 2016). Moreover, the popularity and widespread use of internet also allow consumers to seek information online and reduce the health risks associated with food contamination incidents. The news media and journalists have an important role in reporting on the outbreaks, threat and its cause, including expert commentary regarding the chemical food contaminants. Furthermore, the public need to keep a healthy degree of skepticism about the contaminated food products reported on the news and avoid consuming the accused food products until scientific evidence justifies immediate action. Most importantly, the food industries must accept the need to be more honest and upfront in producing safe commercial food products as well as protecting the public from food contamination.

## Conclusion

The chemical contamination of food has emerged as a serious concern with potential health hazards in their wake. Majority of the food contamination occurs through naturally occurring toxins and environmental pollutants or during the processing, packaging, preparing, storage, and transportation of food. As the technology advances, the detection of such contaminants becomes easier. However, there are several contaminants that are still unknown and research continues in this regard. Although the government has taken adequate steps to minimize the individual exposure to food contaminants, there are still measures that need to be taken to reduce the health risks and diseases that come with the chemical food contamination.

## Author contributions

IR designed, conceived, and wrote the manuscript. WK helped in writing. WP and JL critically reviewed, edited, and finalized the manuscript for submission.

### Conflict of interest statement

The authors declare that the research was conducted in the absence of any commercial or financial relationships that could be construed as a potential conflict of interest. The reviewer AJ declared a shared affiliation, with no collaboration, with one of the authors, IR, to the handling Editor.
